# Ethanol downregulates gastrula gene expression and cell movement, causing symptoms of foetal alcohol spectrum disorders

**DOI:** 10.1242/bio.061777

**Published:** 2025-06-06

**Authors:** Amena Ali Alsakran, Bethany Gibson, Hoi Ying Wong, Caitlin Heaton, Rebekah Boreham, Rashid Minhas, Jonathan Ball, Tetsuhiro Kudoh

**Affiliations:** ^1^ Biosciences, University of Exeter, Exeter EX4 4QD, UK; ^2^ Biology Department, Prince Sattam Bin Abdulaziz University, Al-Kharj, Saudi Arabia

**Keywords:** Zebrafish, Foetal alcohol spectrum disorders (FASD), Ethanol, Alcohol, ACQUIFER

## Abstract

Foetal alcohol spectrum disorders (FASDs) occur in embryos when they are exposed to maternally supplied alcohol. To study the mechanisms of FASDs, the zebrafish embryo can serve as an excellent model as ethanol-exposed zebrafish embryos exhibit common symptoms of human FASDs including microcephaly, incomplete neural plate closure, eye defects, craniofacial disorders and many other defects. Here, we investigated the embryo development at gastrula stage where three germ layers develop with specific gene expressions and undergo dynamic cell movement including extension, convergence and epiboly, establishing the platform to form the head and body axis in later development. Gastrula cell movement analyses using fluorescent transgenic zebrafish embryos revealed that ethanol induced dose-dependent delay of extension, convergence and epiboly cell movement and associated gene expression in all three germ layers. Our results suggest multiple targets of ethanol including gene expression and cell movement, which, consequently, delay key gene expression and cell localisation, causing irreversible developmental defects in the head and body axis formation.

## Teaser

**Summary:** Ethanol alters gene expression and cell movement at the gastrula stage in zebrafish embryos.

## INTRODUCTION

Foetal alcohol spectrum disorder (FASD) is a term used to represent a spectrum of birth issues resulting from the mothers' alcohol consumption. Approximately 9.8% of pregnant women worldwide consume ethanol, with a reported prevalence of FASD of 4.4 per 1000 among children born in the USA (Popova et al., 2019). Multiple FASD-related abnormalities can occur in the fetus' central nervous system (Wilhelm and Guizzetti, 2016), such as microcephaly, incomplete neural tube closure, mental retardation and blindness with suppressed retinal development.

FASD research largely relies on translational animal models, such as rodents and zebrafish, to discover the mechanisms of toxicity of ethanol (EtOH) in embryonic and foetal development (Pinheiro-da-Silva and Luchiari, 2021). The zebrafish embryo has been an ideal model for investigating various angles of ethanol toxicity, including cellular and genetic toxicity and toxic responses at the organism level (Alsakran and Kudoh, 2021). Features such as embryo transparency, time-efficient analyses, high fecundity, short life-span, and toxicity specific biosensor transgenic technology, and availability of genetic data favour their application in toxicity assessment studies (Lee et al., 2012; Mourabit et al., 2019). Ethanol-exposed zebrafish embryos exhibit pre-hatching and post-hatching growth deficiencies, which are similar to FASDs in children (Arenzana et al., 2006). Ethanol negatively affects various aspects of the zebrafish life cycle in a dose-dependent manner, which includes disturbed gastrulation cell movement, embryonic development, cell death, alteration in stem cell gene expression, microcephaly, CNS morphogenesis, neuronal development and skeletal dysmorphogenesis (Alsakran and Kudoh, 2021; Carvan et al., 2004). Retinal cell differentiation and proliferation were also affected in response to ethanol exposure (Fu et al., 2021). Multiple studies have reported ethanol toxicity-based defects in the early stages, which include delayed epiboly resulting from abnormal cell adhesion and movement (Sarmah et al., 2013; Alsakran and Kudoh, 2021). Marrs et al. (2010) reported a reduction in embryo body length in addition to epiboly delay, while Sarmah et al. (2020) revealed the disruption of the microtubule cytoskeleton that restricts the formation of microtubule filaments, which are crucial for epiboly movement during gastrulation. Although cell movement and gene expression are both affected by ethanol at the gastrula stage, it remains unknown if these effects are mutually linked with an epistatic relationship. It is also unclear how these early defects at the gastrula stage specifically cause later morphological abnormalities such as a small brain, small eyes, open brain and other morphological abnormalities. In this project, we analysed gastrula cell movement in detail using a large number of samples with the Acquifer multi-well time-lapse imaging system, using fluorescent transgenic zebrafish lines Tg(h2a:gfp) and Tg(gsc.gfp), which are suitable for visualising the morphology of blastoderm and axial mesoderm, respectively. We have also analysed key gene expression at the gastrula stage in all three germ layers – endoderm, mesoderm and ectoderm – which play a crucial role in the following embryonic morphogenesis. By combining these results, we discuss the toxicity mechanisms of EtOH while linking gastrula stage cell movement, gene expression and following development and body patterning.

## RESULTS

### Live imaging of wild-type (WT) zebrafish embryos treated with EtOH reveals dose-dependent morphological abnormalities and lethality

**Fig. 1.**
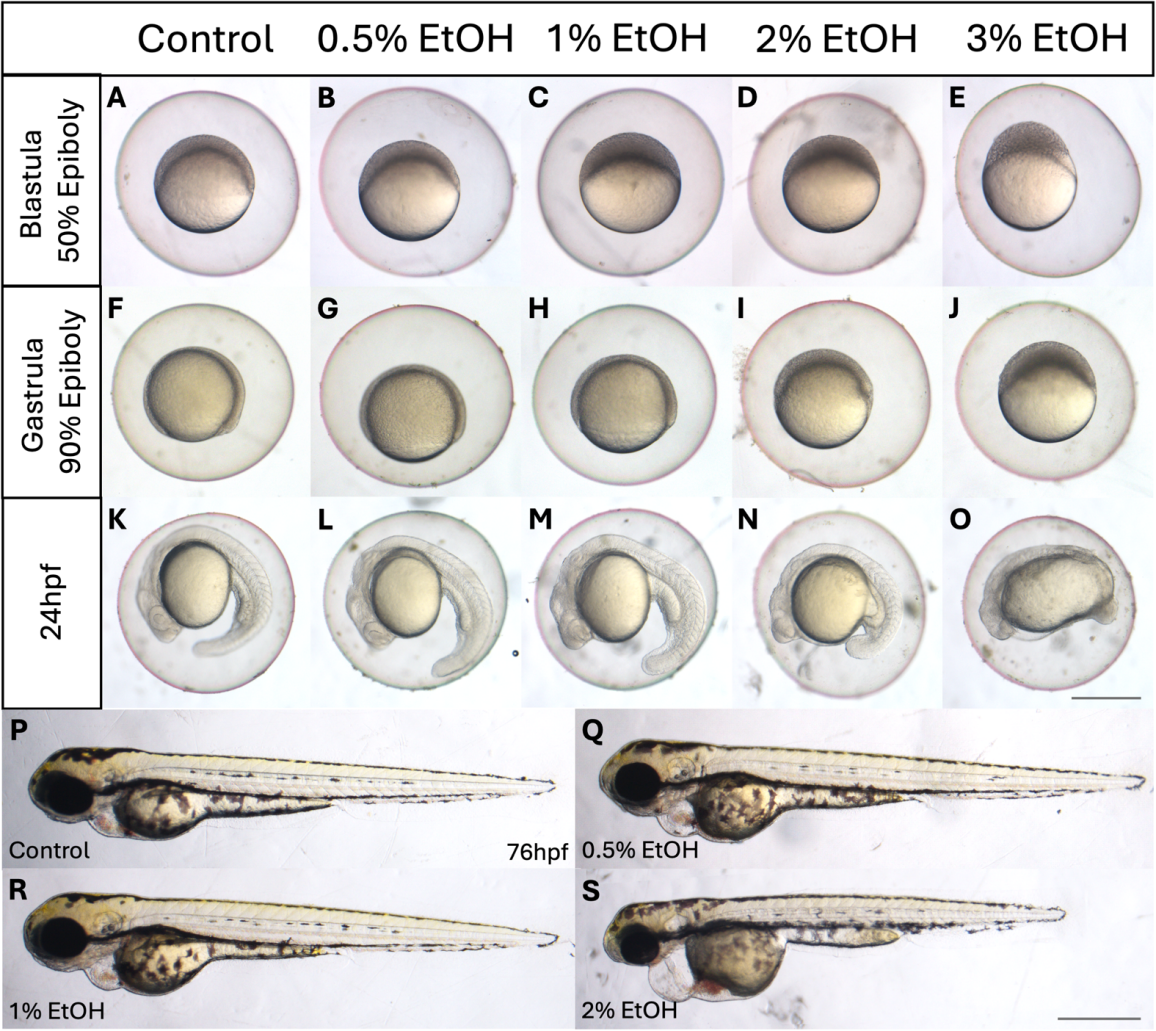
**Morphological defects observed in the ethanol-treated embryos at late blastula (A-E), late gastrula (F-J), 24 hpf (K-O) and 78 hpf (P-S).** Scale bars: 500 µm (A-O), 2 mm (R-S).

To examine the effect of alcohol at early embryonic development around the blastula to gastrula stage, zebrafish embryos were treated with ethanol from 2 hpf (early blastula) with a series of concentrations from 0.5% to 3%. Higher mortality rates were observed with increasing concentrations of EtOH during blastula, gastrula and the following segmentation stages (Fig. 1, Table S1). The highest mortality rates were recorded with 3% ethanol showing a 40% mortality rate for blastula to gastrula and a 66.7% rate for the somitogenesis stages. A 100% survival rate was noted with 0% and 0.5% concentrations for all embryonic stages. Even at the lowest dose of 0.5% EtOH, a degree of deformity was observed at the segmentation stages in the form of oedema in the heart cavity (23.3%), which was also unequivocally noted at 1% and 2% EtOH.

### Fluorescent transgenic zebrafish embryos treated with EtOH show dose-dependent delay of epiboly at gastrula stage

**Fig. 2.**
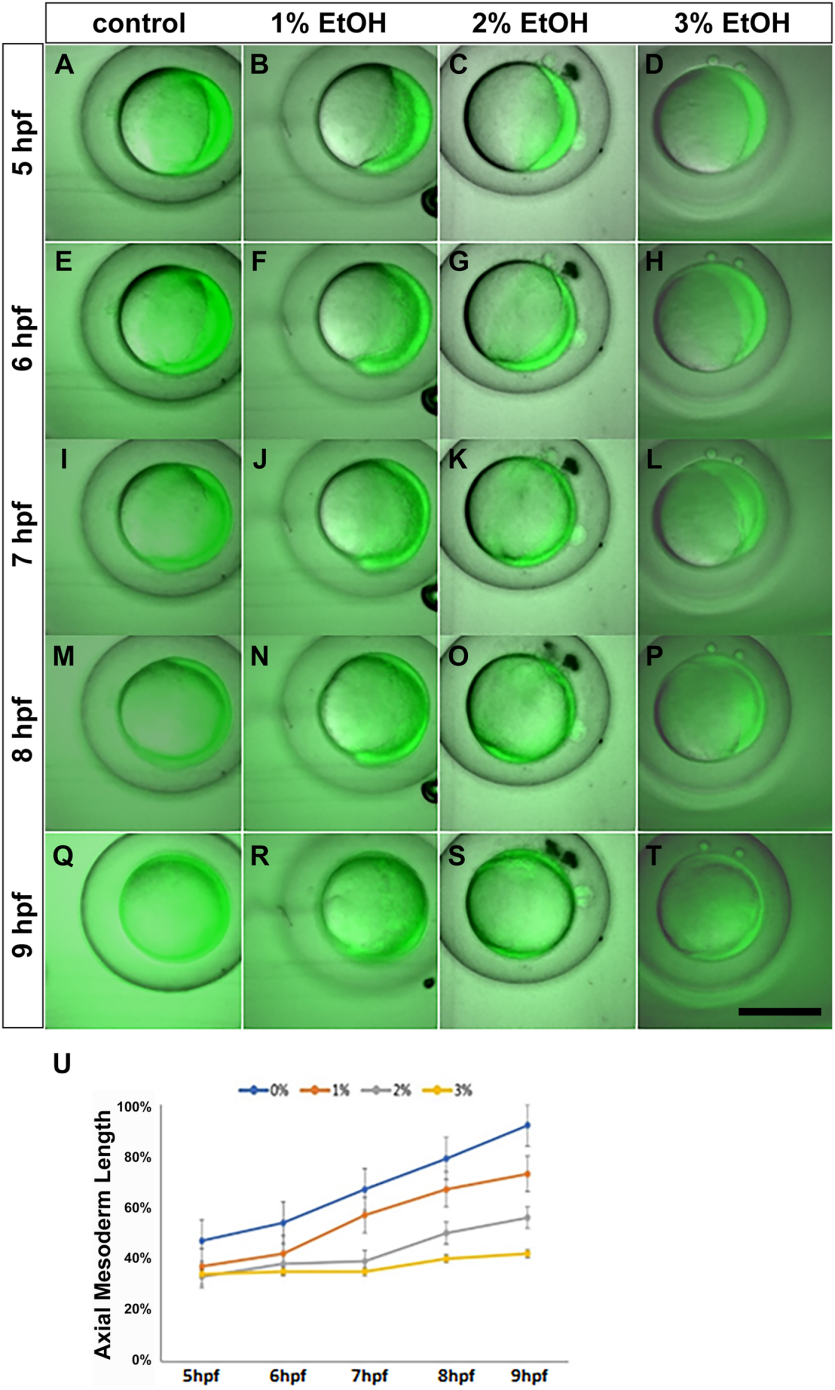
**Tg(*h2a*:gfp) zebrafish embryos reveal dose-dependent delay of epiboly.** The Tg(*h2a*:gfp) embryos shows expansion of the blastoderm during gastrulation. (A-D) 6 hpf, (E-H) 7 hpf, (I-L) 8 hpf, (M-P) 8 hpf and (Q-T) 9 hpf. (U) Measurement of progress of epiboly in the control- and ethanol-treated embryos revealing dose-dependent delay of epiboly by ethanol from 0%, 1%, 2% to 3%. Scale bar: 500 µm.

The transgenic line, Tg(*h2a*:gfp) expresses green fluorescence in a ubiquitous manner in the blastoderm and is suitable for visualising and measuring the progress of epiboly cell movement at the gastrula stage. Therefore, to examine the level of epiboly delay caused by ethanol Tg(*h2a*:gfp) embryos were exposed to ethanol and examined by an Acquifer time lapse imaging device (Fig. 2). There was a noticeable delay in the epiboly movement in the EtOH groups in a dose-dependent manner. The mean epiboly (%) values at 5 hpf remained significantly different from each other (37%, 33% and 34% at 1%, 2% and 3% EtOH concentrations) and from the control treatments (47% at 0% EtOH). There was a gradual delay of epiboly from 5 hpf until 9 hpf. The mean epiboly value of the control (0% EtOH) at 9 hpf was (92%) compared with EtOH treated embryos (73%, 56% and 42% at 1%, 2%, and 3% ETOH concentrations, respectively) (Fig. 2).

### Tg(**gsc**:GFP) zebrafish embryos treated with EtOH reveal delay of extension cell movement at gastrula stage

**Fig. 3.**
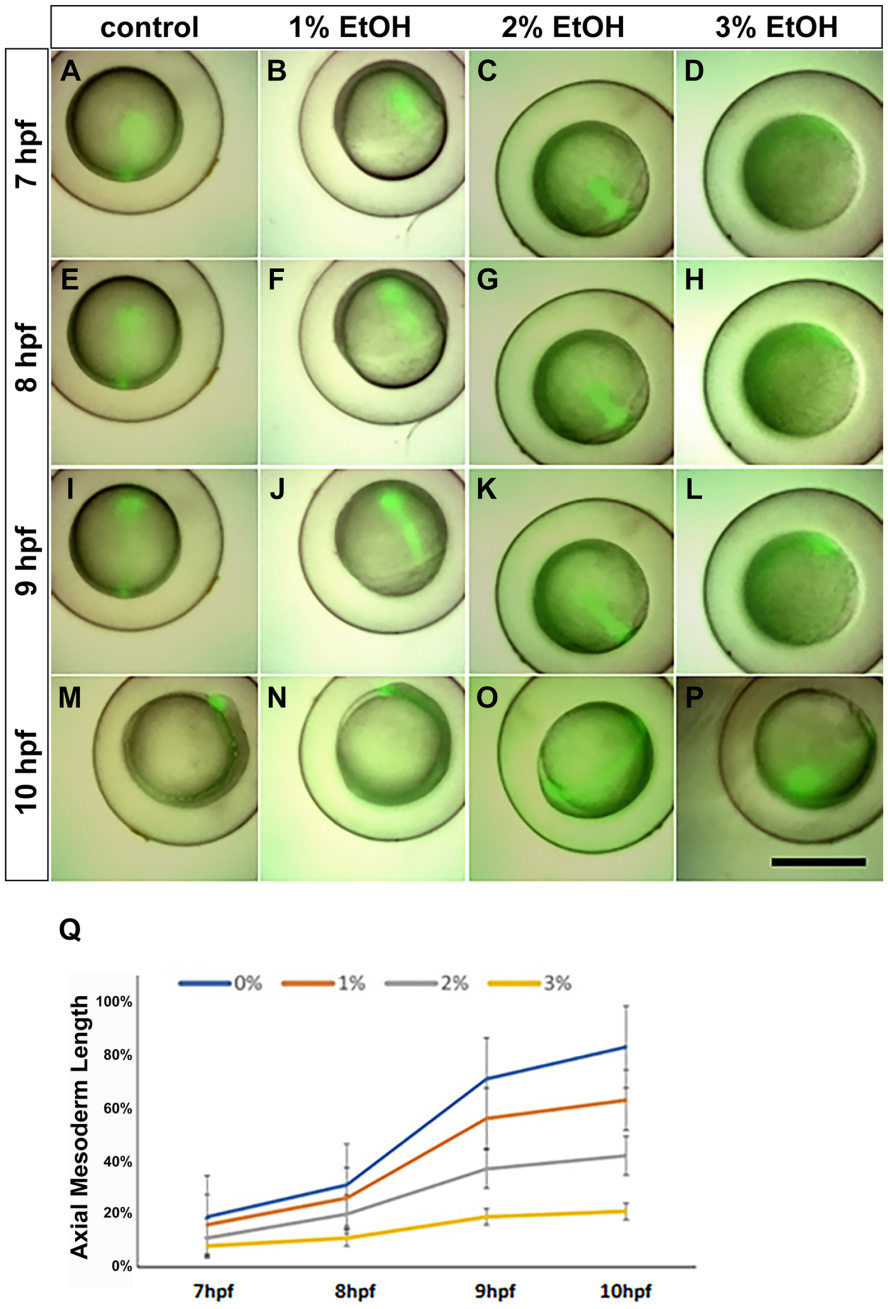
**Tg(*gsc*:gfp) zebrafish embryos reveal dose dependent delay of epiboly.** The Tg(*gsc*:gfp) line shows expansion of the blastoderm during gastrulation. (A-D) 7 hpf, (E-H) 8 hpf, (I-L) 9 hpf, (M-P) 10 hpf. (Q) Measurement of progress of epiboly in the control- and ethanol-treated embryos revealing dose-dependent delay of epiboly by ethanol from 0%, 1%, 2% to 3%. Scale bar: 500 µm.

The Tg(*gsc*:gfp) line marks the axial mesoderm (prechordal plate and notochord) with GFP and is suitable for visualising the extension movement of the axial mesoderm (Boutillon et al., 2022) (Fig. 3). Our data shows that the axial mesoderm extension was delayed by EtOH exposure. At the end of our measurement, the mean growth values at 10 hpf were also found to be significantly different from each other (83%, 63%, 42%, 21% at 0%, 1%, 2%, and 3% EtOH concentrations) (Fig. 3). These data suggest that EtOH also delays the extension cell movement during the gastrula stage.

**Fig. 4.**
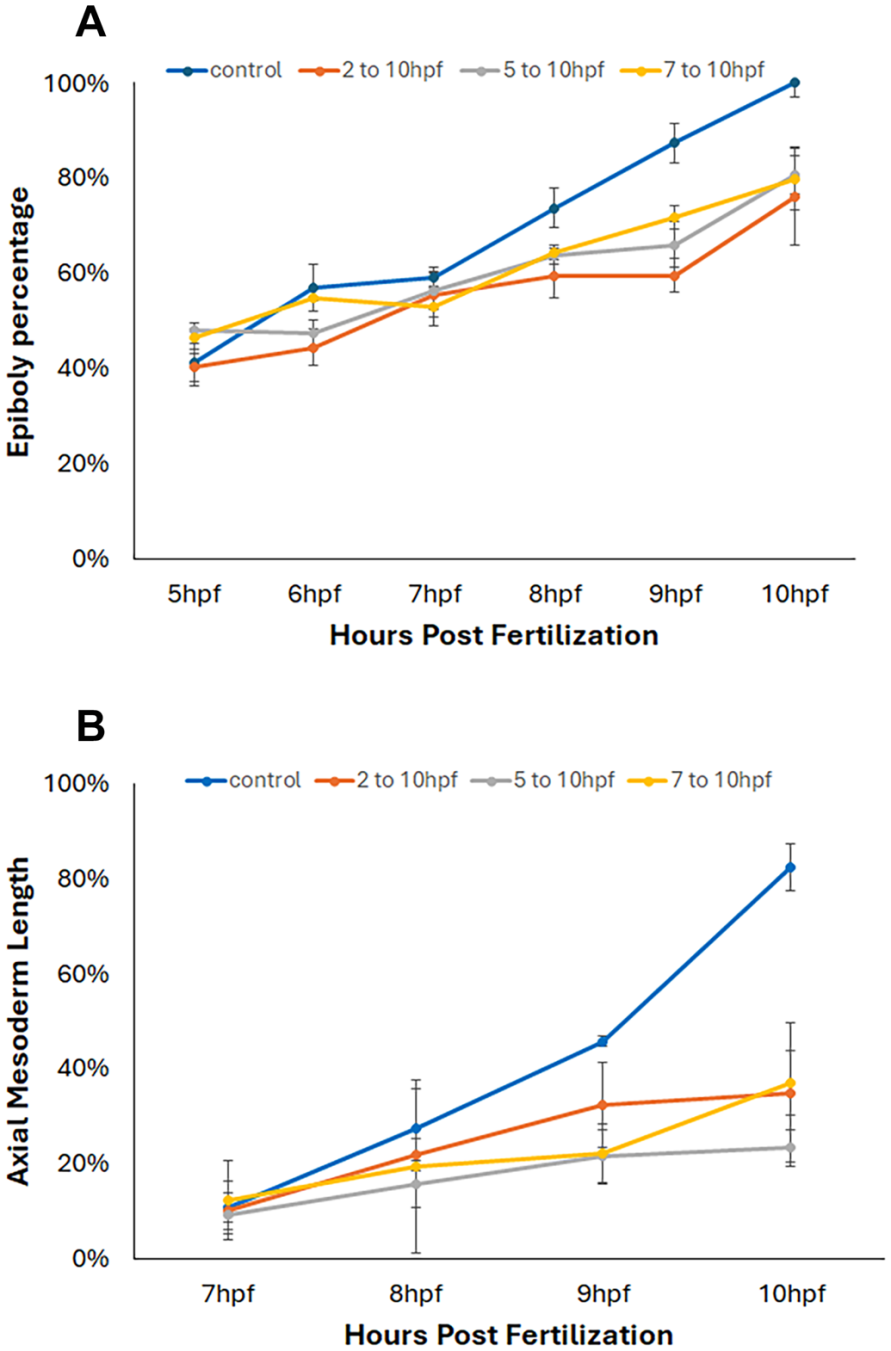
**Timing specific gastrulation cell movement defect induced by ethanol.** Ethanol (2%) was exposed to the zebrafish embryos of Tg(*h2a*:gfp) (A) and Tg(*gsc*:gfp) (B) at different stages (2-10 h, 5-10 h or 7-10 hpf). All ethanol-treated embryos at different timings showed similar cell movement defects.

To examine how quickly ethanol may affect the gastrulation cell movement, we subsequently added ethanol with a selected concentration (2%) at different timing from 2 h (morula), 5 h (late blastula) or 7 h (mid gastrula) using Tg(*h2a*:gfp) and Tg(*gsc*:gfp) embryos, and measured the progress of epiboly and axial mesoderm extension, respectively (Fig. 4). We observed no clear difference between the ethanol-treated embryos at different timings. From these results we concluded that ethanol immediately causes cell movement defects when applied to the embryos.

### Gene expression analyses using *in situ* hybridisation reveal reduction of gene expression in a variety of genes and tissues and delay of the convergence-extension cell movement

**Fig. 5.**
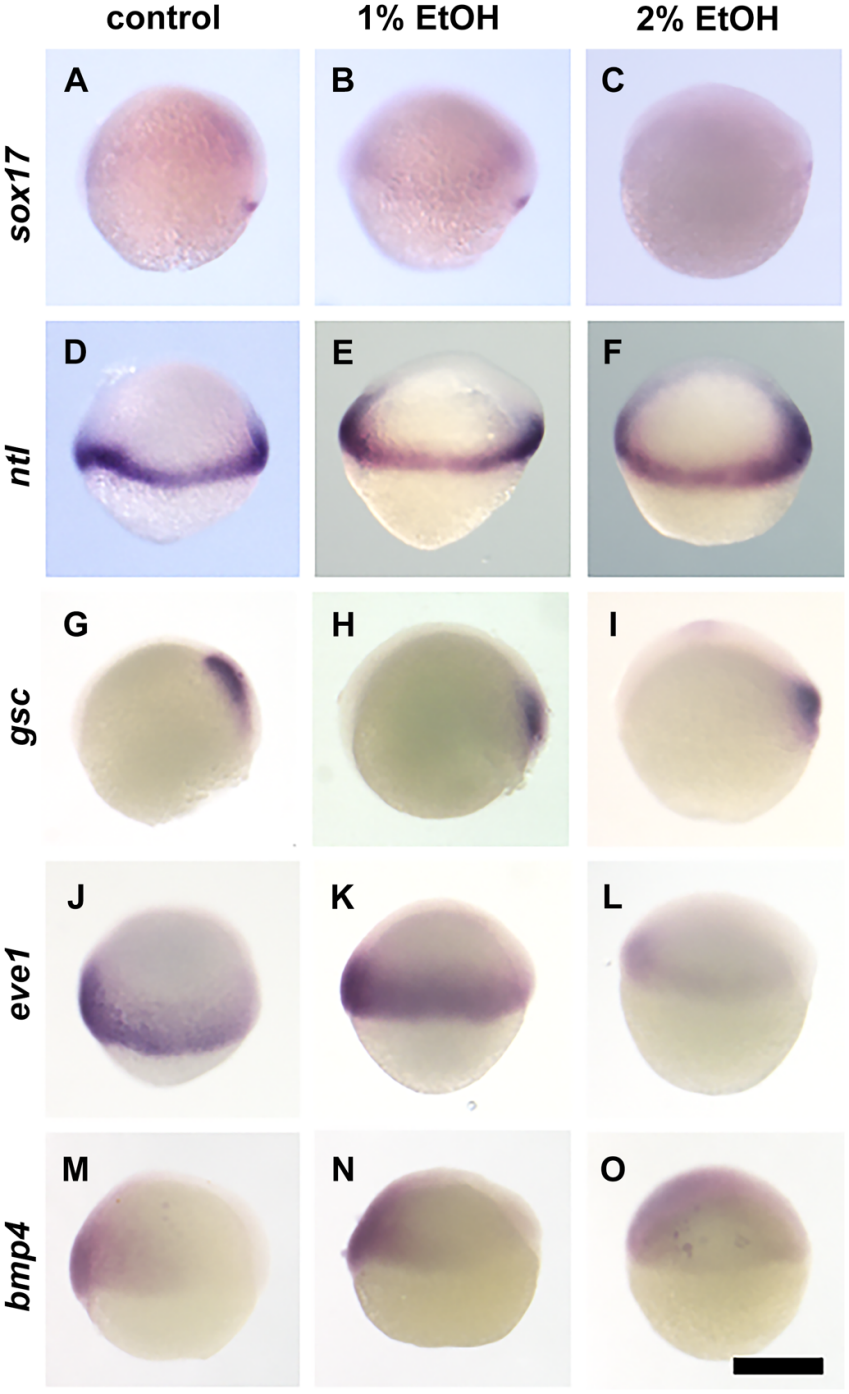
**Expression of endoderm and mesoderm genes are suppressed and/or altered by ethanol.** *In situ* hybridisation staining of late gastrula embryos stained with *sox17* (A-C), *ntl* (D-F), *gsc* (G-I), *eve1* (J-L) and *bmp4* (M-O). Scale bar: 250 µm.

As embryo morphology changed through EtOH exposure, we examined the expression of marker genes for each embryonic domain and cell lineage at the gastrula stage using *in situ* hybridisation. Figs 4 and 5 show the results of markers for endoderm/mesoderm and ectoderm, respectively. In the endoderm/mesoderm markers, there was an overall trend that gene expression was not clearly suppressed by 1% EtOH but the delay of epiboly was visualised by these markers. In addition, convergent-extension movement of endoderm and axial mesoderm cells were reduced. For example, *sox17*, a marker for endoderm, is broadly expressed spreading towards the animal pole (Fig. 5A) (Kudoh and Dawid, 2001), but with the addition of 1% ethanol, the expression domain is further away from the animal pole (Fig. 5B) suggesting that the extension movement of the endoderm layer is delayed. With 2% ethanol, *sox17* gene expression is not clearly visible (Fig. 5C) suggesting the gene expression is suppressed or the development is delayed. *Gsc* is a marker for the axial mesoderm (Kudoh and Dawid, 2001) and the expression domain moves towards the animal pole during gastrulation (Fig. 5G); however with 1% or 2% ethanol, the expression domain stays near the blastoderm margin and more distant from the animal pole (Fig. 5H,I). This confirms that the axial mesoderm extension movement is also delayed as shown in the time lapse imaging of the *gsc*:GFP Tg fish embryos (Fig. 3). *Ntl* is a marker for mesoderm and is expressed in the blastoderm margin (Kudoh and Dawid, 2001). The margin will move towards the animal pole by epiboly cell movement, therefore the *ntl* expression domain is located more closer to the vegetal pole at late gastrula stage (Kudoh et al., 2004). With ethanol, the *ntl* domain is located further away from the vegetal pole (Fig. 5D-F), supporting the idea that epiboly movement was delayed by ethanol.

In 2% EtOH treated embryos, *sox17*, *eve1* and *bmp4* were strongly suppressed (Fig. 5C,L,O) but *ntl* and *gsc* were not (Fig. 5I,L).

**Fig. 6.**
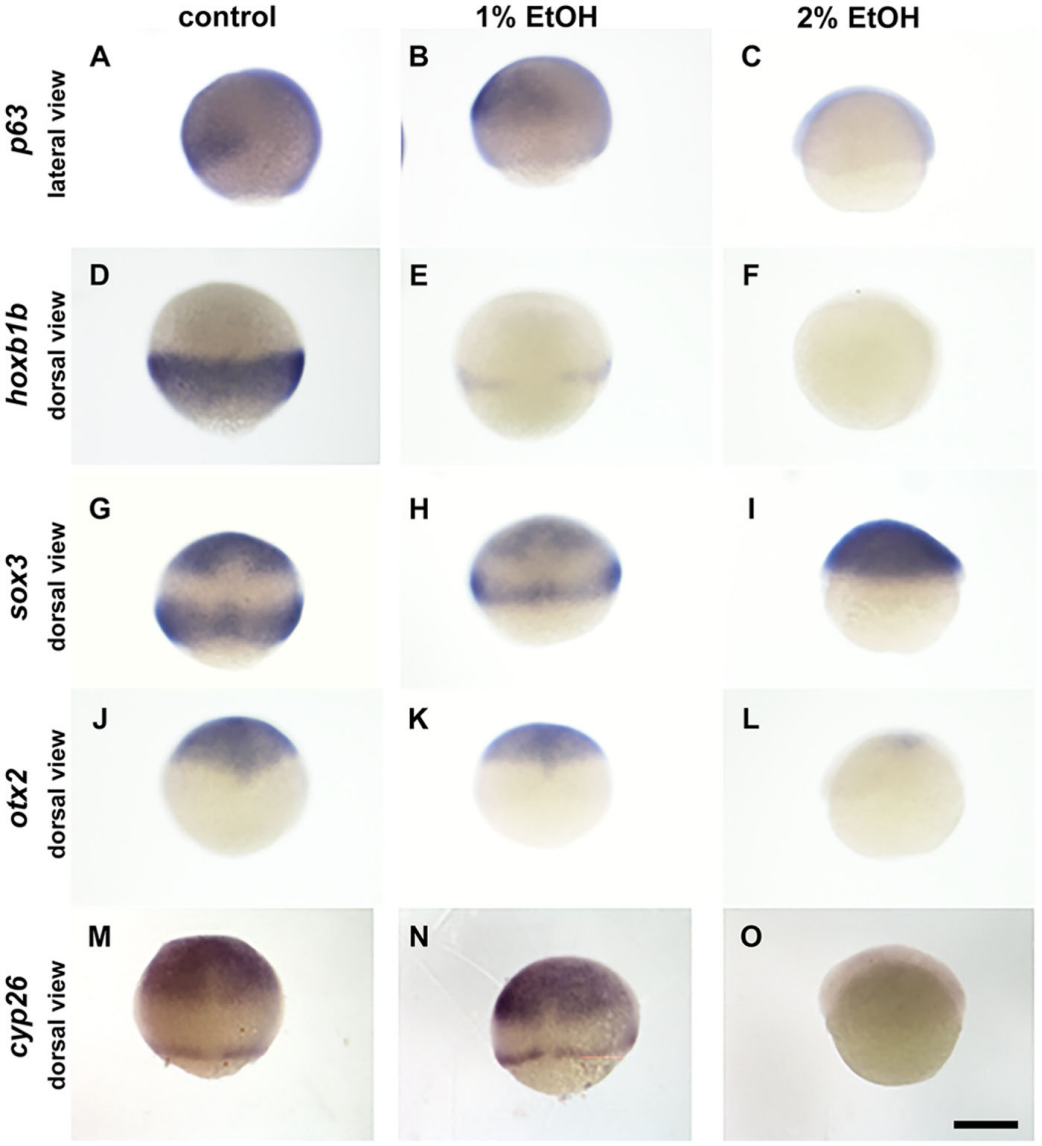
**Expression of ectoderm genes are suppressed and/or altered by ethanol.** *In situ* hybridisation staining of late gastrula embryos stained with *p63* (A-C), *hoxb1b* (D-F), *sox3* (G-I), *otx2* (J-L) and *cyp26a1* (M-O). Scale bar: 250 µm.

Consistently to these results, in case of ectoderm markers, most of marker genes were not clearly suppressed with 1% EtOH and only shows narrowing of expression domains due to the epiboly delay (Fig. 5). However, *hoxb1b* showed narrowing of the expression domain with clear decrease of gene expression (Fig. 6E). With 2% EtOH treatment, as seen in the endoderm/mesoderm markers, most of ectoderm markers were also strongly suppressed except *sox3* (Fig. 6I). Only the ectoderm marker *sox3* was detected during 2% EtOH exposure of the genes investigated.

**Fig. 7.**
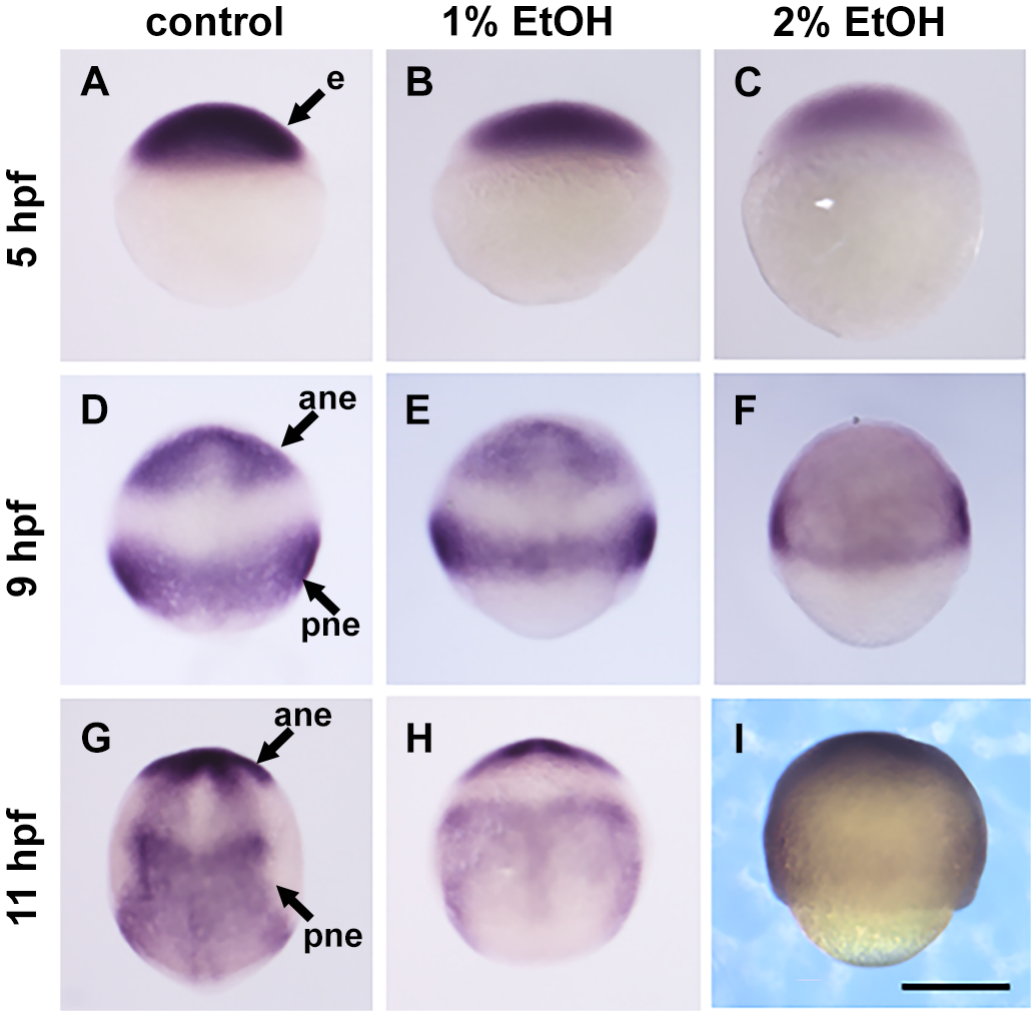
**Expression of neural ectoderm gene *sox3* is reduced or delayed by ethanol in a stage specific manner at blastula to gastrula stages.** (A-C) 5 hpf, (D-F) 9 hpf, (G-I) 11 hpf. e, ectoderm; ane, anterior neural ectoderm; pne, posterior neural ectoderm. Scale bar: 250 µm.

Since *sox3* has complicated expression patterns, further analysis of *sox3* expression was conducted with different developmental stages including late blastula, late gastrula and bud stages (Fig. 7). The data revealed that *sox3* has two waves of gene expression, firstly in the blastula stage, broadly in all ectoderm (Fig. 7A) and subsequently in the neural ectoderm in two domains (anterior and posterior) (Fig. 7D). The stage specific *sox3* expression analysis confirmed that 1% EtOH did not affect the gene expression but reduced the convergence extension cell movement. On the other hand, in the embryos exposed with 2% EtOH, *sox3* gene expression level seems lower in all domains examined and a more severe delay of epiboly movement was observed.

## DISCUSSION

In this work, we used the zebrafish embryos as a model for studying human FASD focusing on gene expression and cell movement during gastrulation. After ethanol exposure with different concentrations, microcephaly, reduction of body length and eye size, pericardial oedema and enhanced mortality are induced as reported from similar exposure from previous reports (Bilotta et al., 2004; Sylvain et al., 2010; Soares et al., 2012; Joya et al., 2014).

To elucidate the cause of these symptoms, we conducted time-lapse live imaging analyses using an automated multi-well imaging system for measuring gastrulation cell movement including convergence (cell movement from ventro-lateral towards dorsal), extension (cell movement along animal-vegetal poles) and epiboly (expansion of the blastoderm towards the vegetal pole). To clearly visualise the progress of epiboly, Tg(*h2a*:gfp) transgenic line, which ubiquitously expresses GFP in the embryonic cells, was used (Fig. 2). These data indicated a dose-dependent delay or end of epiboly by EtOH.

The level of delay was partially restored at around bud stage with an intermediate dose of EtOH (e.g. 1% and 2%), suggesting that some level of delay can be amended, and normal morphological development could still be achieved with a low to medium dose of ethanol at least for the epiboly-mediated developmental processes. However, with higher dose of EtOH (2% and 3%) epiboly delay became more severe, causing incomplete epiboly, failure of the germ ring to close, and the blastoderm failing to cover the yolk, causing embryonic lethality during gastrula stage. Epiboly cell movement is a relatively flexible process during development, and some delay can possibly be restored. In zebrafish, epiboly is complete before the bud stage, then the tail bud is formed and somitogenesis follows. However, in medaka, epiboly is complete at the beginning of the somite stage (Iwamatsu, 2004). In a more extreme case in rainbow trout, completion of epiboly delays to the mid-somite stage (Finch et al., 2010). These differences suggest that epiboly delay may not directly affect other essential gross developmental processes such as head development, somitogenesis and tail bud formation. However, when the epiboly delay became more severe at higher doses of EtOH (2% and 3%), the delay and abnormalities were not fully restored, leading to stopping of epiboly, incomplete gastrulation, and failure of the germ ring closure. Such severe phenotypes tend to cause failure of the complete tail bud formation leading to a short and disorganised tail structure (e.g. Mourabit et al., 2014). It has been reported that ethanol affects the microtubule cytoskeleton in the yolk syncytial layer resulting in the suppression of microtubule filament production, which is crucial for epiboly migration (Sarmah et al., 2020; Takesono et al., 2012); therefore, such molecular mechanisms might be the cause of the epiboly delay.

According to Blader and Strahle, 2.4% ethanol exposure at the gastrula stage inhibited the extension of the axial mesoderm, resulting in the failure of optical field separation and cyclopia (Blader and Strähle, 1998). Besides Tg(*h2a:gfp*), we have also used Tg(*gsc:gfp*) fish line for multi-well live imaging. In this line, GFP is expressed in the axial mesoderm, including in the prechordal plate and notochord, and is suitable for measuring the extension movement of these tissues. These data show dose-dependent delay of the extension and failure of the axial mesoderm reaching to the animal pole. As seen in the delay of epiboly, the delay of axial mesoderm extension with a mild ethanol dose (1%) seems to be restored at the end of gastrula to early somite stage; therefore, the body size and morphology at 24 and 48 hpf in 1% EtOH is similar to the control. Consistent with this, eye size and eye separation are relatively normal at 1% EtOH, but in contrast, at a higher dose of EtOH, eye size is reduced, cyclopia and microcephaly are induced, and these phenotypes seem to be correlated with the delay of the failure of the axial mesoderm reaching towards the animal pole.

Although cell movement may explain many phenotypes of EtOH exposure, it is also important to examine gene expression as such changes can also alter cell movement, cell differentiation, cell proliferation and consequently control tissue development and embryo morphogenesis. We have tested markers for the three germ layers, endoderm, mesoderm and ectoderm. The overall pattern demonstrates that with 1% EtOH gene expression was not suppressed except in *hoxb1b*. However, with 2% EtOH, gene expression was mostly suppressed except in *ntl* and *gsc*, which were less affected in terms of gene expression level. Cell movement including epiboly, convergent-extension movement were all highly suppressed at 2% EtOH, as seen in all germ layers markers including endoderm (*sox17*), axial mesoderm (*gsc*) (Fig. 4) and ectoderm (*p63, sox3*, Figs 5 and 6).

Overall, reduction and/or delay of gene expression occurred in all germ layers. This suggests that the transcription process in general was possibly a target of alcohol toxicity and the efficiency of the production of mRNAs was reduced.

Further analyses to investigate timing specific effect of EtOH on gastrulation cell movement, 2% EtOH was applied at 5 h (end of blastula) and 7 h (mid gastrula) in the Tg(*h2a*:gfp) and Tg(*gsc*:gfp) embryos. In both stage-dependent treatments, it showed similar delay of epiboly and extension cell movement as continuous exposure from 2 hpf (Fig. 4). This suggests that the effect of EtOH on cell movement is very quick and direct, therefore it does not need to involve gene expression changes. It is known that epiboly defects tend to reduce convergent-extension cell movement. However, if epiboly is the primary defect, the defect in the convergent-extension movement shows a milder phenotype (Kane et al., 2005; Takesono et al., 2012). In the case of EtOH, both epiboly and C-E movement showed an equally severe phenotype suggesting both cell movements are equally affected by EtOH and direct targets of toxicity.

To explain the microcephaly, reduction of the head neural ectoderm markers, *otx2* and *cyp26a1* seems important (Fig. 5). The area to be specified as head neural ectoderm (anterior neural ectoderm) is defined by the balance of bmp, bmp-angonists (e.g. chordin) and posteriorising activities (wnt, fgf and retionic acid) (Kudoh et al., 2002, 2004). Therefore, it is possible that these signalling activities are also compromised in the EtOH treated embryos, and consequently head neural induction is reduced and/or delayed.

*Sox3* is broadly expressed in the neural ectoderm and its expression demonstrated a particularly complex pattern of response to EtOH. At 8 hpf (80% epiboly for control), control embryos exhibited two expression domains, anterior neural ectoderm (animal-dorsal spot) and posterior neural ectoderm (band close to the blastoderm margin), however, embryos with 2% EtOH showed higher gene expression at this stage broadly spreading in the ectoderm without having two distinct expression domains (Fig. 5). However, by carefully examining earlier and later stages with and without EtOH, we found that the first wave of gene expression occurs from late blastula to early gastrula with broad ectodermal expression and the second wave of *sox3* expression occurs at mid to late gastrula stage in the two domains (anterior and posterior). By seeing these two steps of the *sox3* gene expression we concluded that the first wave of *sox3* expression is delayed by EtOH and the second wave of the *sox3* expression is strongly reduced with 2% ethanol.

Among all marker genes tested, the most highly suppressed gene by EtOH was *hoxb1b*. This gene is a marker of the posterior neural ectoderm (prospective hindbrain and spinal cord) (Fig. 7). All other genes were only slightly reduced at 1% ethanol, but only this gene was highly reduced even at 1% EtOH and fully downregulated at 2% EtOH. The strong reduction of the *hoxb1b* expression seems to be caused by two mechanisms. Firstly, *hoxb1b* is induced by retinoic acid which is generated and secreted from the involuting paraxial mesoderm (Kudoh et al., 2002). If involution of paraxial mesoderm is delayed, *hoxb1b* expression domain would become narrower and expression would become weaker. Secondly, in most of genes transcription event seems lower in the EtOH-treated embryos. Therefore, in case of *hoxb1b*, these two effects by EtOH, cell involution movement and signalling, and general reduction of transcription may have caused additive suppression of the gene expression.

*ntl* and *gsc* were the most unaffected by EtOH in terms of gene expression level (Fig. 5). The resistance to EtOH for these genes may be due to the fact that these are the earliest marker genes being expressed from mid blastula stage. All other markers are only expressed at late blastula to early gastrula stages. *ntl* and *gsc* are induced by the *Sqt* nodal signalling pathway (Kudoh and Dawid, 2001) and signalling molecules of this pathway are already equipped from maternal storage in the egg. Therefore, it is possible that signalling was not largely affected.

Overall, our data revealed that all cell movements that we tested (convergence, extension, epiboly) are reduced by EtOH, and all genes expression are also reduced at some extent in a dose-dependent manner. Some gene expressions are more severely affected by EtOH possible due to the combinatorial effects of transcription regulation and indirect consequence of cell movement and signalling.

EtOH exposure in the first 24 h of zebrafish development (including gastrulation phases) is equivalent to EtOH exposure in the third week of human pregnancy (Liu and Lewis, 2014; Wallén et al., 2021). It is highly conceivable that exposure of human foetus to EtOH would cause similar effect on both cell movement and gene expression in a human foetus. In humans, it has been thought that 0.08% or above of blood alcohol level may cause FASDs. In case of zebrafish, roughly 30% of water alcohol level is incorporated into the zebrafish embryo (e.g. 1% EtOH in water cause 0.3% EtOH level in zebrafish embryo) (Lovely et al., 2014). Therefore 1% EtOH exposure by water is highly relevant to human FASDs cases, which can cause clear FASDs symptom without causing lethality. On the other hand, EtOH 2% in water can cause severer phenotype in epiboly, gene expression and morphology and can possibly mimic the severe FASDs symptom leading to a lethal phenotype.

There are three reasons conceivable why gastrula stage is highly sensitive to ethanol toxicity. Firstly, it does not have a skin layer, therefore penetration of ethanol is easier. Secondly, cells are in process of dynamic gastrulation cell movement and this event is the direct target of ethanol (Sarmah et al., 2013). And finally, many detoxifying enzymes and signalling pathways are not yet established at gastrula stage, therefore cells at this stage are more vulnerable to toxic chemicals compared to the 24 h and following stages (Kobayashi et al., 2002).

Our multi-well automated imaging assay system for examining the progress of epiboly is a very quick and high-through put assessment for it. In this system, embryos are randomly oriented but by calculating the angle of the blastoderm margin (see Materials and Methods), we were able to measure the epiboly with sufficient accuracy. To ensure our measurement is accurate enough, we have omitted some samples for which the orientation was wrongly angled (animal pole or vegetal pole facing to the camera). The large *n* number in the 96-well system further supported the accuracy of the live epiboly measurement. A reliable and quantifiable system to determine the effect of ethanol on gastrulation would also allow the quick and efficient screening and assessment of drugs and nutrients that can reduce the toxicity of EtOH using this automated imaging system.

## MATERIALS AND METHODS

### Zebrafish (*Danio rerio*)

Zebrafish strains, wild Indian karyotype (WIK), Tg(*h2a*:GFP) (Pauls et al., 2001) and Tg(*gsc*:GFP) (Doitsidou et al., 2002) lines were maintained at the University of Exeter's Aquatic Resource Centre (ARC) supplied with aerated and circulated freshwater dissolved with artificial sea salt (0.2 ppt), 28°C±1°C. Zebrafish eggs were collected by natural spawning in the morning within 30 min after fertilisation. The embryos were incubated at 28°C in Petri dishes.

### Ethanol treatment

Zebrafish embryos were treated with different concentrations of ethanol (0.5%, 1%, 2% and 3%) starting from 2 hpf either in 10 cm Petri dish for live imaging or sampling for staining, or in 96-well plate for Acquifer automated time-lapse imaging. Live images of embryos were captured using Nikon SMZ1500 stereomicroscope. The images were analysed by measuring epiboly and extension of the axial mesoderm. Embryos were randomly oriented in 96-well plates; therefore, the progress of epiboly was calculated by estimating the distance between the animal pole and the plane of the blastoderm margin. The statistical differences among treatments were established by subjecting the data to one-way ANOVA, and means were compared using Tukey's multiple comparison test at *P*<0.0001.

### Automated time lapse imaging and analyses of gastrula cell movement

Control- and EtOH-treated zebrafish embryos with transgenic backgrounds, Tg(*h2a*:gfp) or Tg(*gsc*:gfp) were incubated in a 96-well plate at 28°C from 4 hpf to the end of gastrulation. During this period, both transmission light and fluorescent images with GFP filter (488 nm) were captured every hour. Using FIJI ImageJ, the progress of elongation of blastoderm margin and extension of the axial mesoderm were measured. From the image of Tg(*h2a*:gfp) embryos, the oval of the blastoderm margin was identified and the distance between the centre of the oval and the animal pole was measured to calculate the length of the blastoderm. As for the extension of the axial mesoderm, length of the GFP-positive axial mesoderm structure in the Tg(*gsc*:gfp) embryos were measured. The measurement method is described in detail in Fig. S1.

### *In situ* hybridisation

Zebrafish embryos were fixed with 4% PFA in PBS with chorion and manually dechorionated in PBS with tweezers. Subsequently embryos were stored in methanol at −20°C. Preparation of probes and *in situ* staining methods in zebrafish were conducted as previously reported (Kudoh et al., 2004).

### Ethical declaration

All experimental methods were approved by the University of Exeter ethical committee, UK Home Office animal project license and carried out in accordance with these guidelines.

## Supplementary Material

10.1242/biolopen.061777_sup1 Supplementary information
