# Supplementary material for: Ethanol downregulates gastrula gene expression and cell movement, causing symptoms of foetal alcohol spectrum disorders
Source: Biol Open. 2025 Jun 6;14(6):bio061777. doi: 10.1242/bio.061777 (PMC12171575; doi:10.1242/bio.061777)
Supplement: Supplementary information [file biolopen-14-061777-s1.pdf]

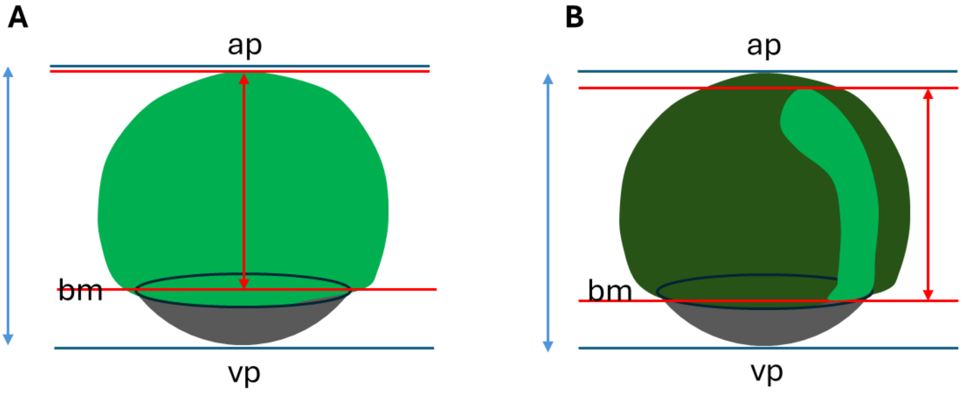

Fig. S1. Measurement of the epiboly and axial mesoderm. **A.** Progress of epiboly was measured using Tg(*h2a*:gfp) zebrafish embryos by measuring the blastoderm length, the distance between the central point of the blastoderm margin and the top of the animal pole (A, red arrowed line). The percentage of epiboly progress is calculated by the ratio of the blstoderm length against the vertical length of the embryo (A. blue arrowed line). **B.** Progress of the axial mesoderm extension was measured using Tg(*gsc*:gfp) zebrafish embryos by measuring the vertical length of the axial mesoderm (red arrowed line). The percentage of the progress of axial mesoderm extension was calculated by the ratio against the vertical embryo length (B. blue line). ap; animal pole, bm; blastoderm margin, vp; vegetal pole.

Gene markers	0%	1%	2%	Abnormal phenotype
sox17	0/11	7/9	7/7	Reduced gene expression
ntl	0/12	9/9	7/7	Delay of movement of the gene expression domain
gsc	1/8	6/8	9/18	Delay of movement of the gene expression domain
eve1	1/13	9/9	8/5	Reduced gene expression
bmp4	0/6	9/9	10/10	Reduced gene expression

Fig. S2. *In situ* staining phenotype and number of zebrafish embryos showing such phenotype and total embryo used for each staining. See embryo phenotype in the Fig. 5.

Gene markers	0%	1%	2%	Abnormal phenotype
p63	0/5	5/5	5/5	Reduced gene expression
hoxb1b	0/4	5/5	5/5	Reduced gene expression
sox3	0/5	5/5	5/5	Delay of stage specific gene expression
otx2	0/7	5/5	5/5	Reduced gene expression
cyp26	0/6	2/3	4/4	Reduced gene expression

Fig. S3. *In situ* staining phenotype and number of zebrafish embryos showing such phenotype and total embryo used for each staining. See embryo phenotype in the Fig. 6.

Gene markers	0%	1%	2%	Abnormal phenotype
sox3 (5hpf)	7/0	8/11	15/21	Delay of stage specific gene expression
sox3 (9hpf)	0/11	8/8	8/9	Delay of stage specific gene expression
sox3 (11hpf)	0/8	10/10	6/6	Delay of stage specific gene expression

Fig. S4. *In situ* staining phenotype and number of zebrafish embryos showing such phenotype and total embryo used for each staining. See embryo phenotype in the Fig. 7.

Table S1. Live embryos phenotype and number of embryos showing the phenotype with ethanol treatment from the experiment shown in the Fig. 1.

Treatment	Total	abnormal	Abnormality phenotype
Control	26	1	
0.5%	26	10	Flat face, curved body
1%	29	11	Flat face, curved body
2%	24	24	Fiat face, short body, lethality
3%	No survival	-	lethality
